# Electro-Physiology, Etc.

**Published:** 1877-12

**Authors:** 


THE following article, written by Dr. A. MEANS, of Oxford, Ga., on Electro-Physiology, is copied from the *Southern Medical Journal* for March, 1848, then edited by the late Paul F. Eve, Augusta, Ga. The views announced by Dr. Means are reproduced by us now, that physiologists may determine to what extent the theories then advanced on the subject by this learned chemist have been verified by subsequent investigations:

---

### ELECTRO-PHYSIOLOGY.

### THOUGHTS UPON THE ADAPTATION OF THE ANIMAL ORGANISM IN THE GENERATION AND MAINTENANCE OF THE ELECTRO-VITAL CURRENTS, Etc.

---

The theme which has elicited the present dissertation, together with the tenor of arguments adopted in its discussion, has already, under another form, met the public eye. But the subject having been regarded of sufficient interest to the profession to entitle the views there advocated to a place in the pages of the *Journal*, I have consented to furnish them for publication, together with such other additional reflections as the very limited time at my command will allow me to supply. It is due perhaps to myself, as well as to the scientific and practical character of the organ through which this communication is now presented, to entreat for it an attentive and unprejudiced perusal, and to express the hope that whether the views which it inculcates be adopted or rejected, they will not, after such examination, be regarded as the mere visionary speculations of a prurient and ambitious philosophy, which constructs its airy fabrics without the supports, and beyond the region of facts, but as the patient deductions of sober reason, purporting, at least, to be drawn from ample experiments, and from the known operation of recognized and established physical laws, some of them, it is true, at first sight, novel and startling in their character, but nevertheless, demanding the confidence of the honest and intelligent enquirer after truth.

Our undisguised conviction of the growing and valuable importance of electro-physiology to the medical philosopher as well as the practicing physician, must be our apology for so earnestly inviting professional attention to the subject.

Since the *body* of the arguments here presented were drawn up in their present form, it is proper to remark that the elaborate and scientific researches of Professor Matteucci, of the Pisa* University, embodied in "Lectures on the Physical Phenomena of Living Beings," has reached me, and to which I will occasionally take the liberty to refer. And although the views of this profound physiologist may not in all their *details*, harmonize precisely with those which are respectfully maintained in the present article, it is unaffectedly gratifying to find the *great fundamental* doctrine here defended, unequivocally announced, as the only reasonable conclusion to be drawn from his laborious and critical experiments upon animal nature. I allude to the generation of electric currents in the human body as the functional result of certain portions of its organism. His language is: "It would be absurd to suppose that the chemical actions of living beings, all of which develop heat and often light, would not be accompanied by the production of electricity." From his experiments he says, "the existence of an electrical current in the muscles has been well demonstrated, and its principal laws are established." An opinion to which we ourselves have been recently committed before an intelligent class, he fully sustains, viz: that "Galvani's assumption" (advocating the existence of animal electricity, and generally supposed to have been disproved by the after experiments of Volta, etc.) "was not erroneous, since a great number of facts discovered by him are certainly due to the electricity *developed in*, and *proper to*, animals."

While it might under other circumstances constitute a subject of interesting enquiry to trace the triumphant progress of the science of chemistry through the last half century, and individualize its immense contributions to our profession and to the world at large, the object had in view in this essay will limit us to the examination of a *peculiar* class of facts (to which allusion has already been made in the foregoing remarks) disclosing new and important principles which deservedly command the increased attention of the philosophic world at the present moment, and destined as we believe, to effect signal revolutions in the psycological as well as physiological views which have heretofore obtained among the ranks of the learned.

---

* In Italy, founded in 1838.

To the investigations of chemical philosophy have been assigned the laws and properties of that invisible, potential and universal agent, whose mysterious *modus existendi* has hitherto baffled the penetration of the astutest intellect, but whose illimitable power and ubiquitary presence are so strongly vindicated in the roll of the thunder, the whirl of the water-spout, and the corruscations of the aurora, or in the less startling, but no less interesting modifications of its action, detected in the violent and spasmodic throes of the human muscle—the intensity and brilliancy of the charcoal lights, and the vivid and instantaneous combustion of the metals in the line of a galvanic circle, or in its still more recently recognized forms of terrestrial magnetism, magneto-electricity, and electro-physiology.

To the views, then, which have forced themselves upon our attention in examining this strange and subtle, but effective and pervading, agent in its connexions with the animal economy and the laws of life, we beg leave mainly to confine the investigations which may follow. Nor, in canvassing the topic before us, will our limits allow, (did the reliable data at our control justify it) that detailed survey and elaborate argumentation which a persistent and incorrigible skepticism demand, and which the ever accumulating resources of science shall, at no distant day, triumphantly furnish. The recent ascertainment and novelty of many of the facts to be submitted, must, in the present state of our knowledge, necessarily leave *any* theory projected for their solution, embarrassed with unexplained difficulties which time and research can alone dissipate. It is the province of sound philosophy ever to make a clear distinction between *facts* and the *reasons* of them. *One fact* properly authenticated may not be rejected or challenged on the claims of a thousand adverse theories, though the rationale of its origin may remain unexplained for centuries. The very spirit of the Baconian philosophy supposes the previous evidence and patient classification of facts, from which physical laws are to be deduced, and rational theories constructed. And when in the progress of discovery the accumulation of authenticated testimony requires the abandonment of a supposed law, or the renunciation of a favorite theory, candor, conscience, and science demand at our hands its prompt surrender. But when the field of observation is large, interesting, and comparatively unexplored, with only a few well defined and settled points within the range of intellectual vision, and these, in their relationships and bearings, open to the enlightened conjecture, *theory*, cautiously adopted, is not only admissible, but by systematizing our views, stimulating enquiry, and directing experiments, is powerfully instrumental in evolving truth and enlarging the boundaries of knowledge. Such, precisely, are the conditions which surround us when attempting to prosecute our enquiry into the subtle and recondite causes of vital phenomena by the aid of the guiding lights which electricity and the organic sciences hold up to our view. In former ages this whole interesting region lay profoundly submerged in the deeps of popular and professional ignorance. All was one wide waste of waters where the ark of speculation floated on in its devious and doubtful course. But time has made its steady progress, the deluge has begun to assuage, and the pioneer dove which had once and again been despatched by philosophic enquiry, only to return on drooping wing from her cheerless flights, has at length appeared with the olive leaf in her beak. Summit after summit now peers above the flood, until a hundred lofty peaks are glowing in the sunshine of science and the ark is seen settling on the Ararat of truth. And although the physiologist of the present age cannot define all the connexions which subsist between these bright elevations, that lie like sunlit islands upon the retiring seas, yet the waters will have soon found their lowest level, and their beautiful natural relationships will be distinctly traced to the great *fundamental range* of *physical laws*, which sustain and characterize the whole organism.

But more directly to the execution of my purpose. So uniform and harmonious is nature in the maintenance and execution of her laws, that by the aid of observation and analogy, we may generally determine with tolerable precision the habitudes, functions or phenomena of bodies, from their position, conformation or structural arrangement. With such inimitable exactitude are these laws observed that a *single* fossil bone, exhumed from its isolated bed, by the unerring physical characters which it bore, enabled the learned and sagacious Cuvier to read its natural history—mentally to reconstruct the whole skeleton, and to classify the original animal with the genus and species to which it belonged. Indeed but for these invaluable indications, our researches in comparative anatomy, physiology, hygiene, mineralogy, geology, and the kindred sciences, must be seriously impeded, if not finally arrested. In view of this pervading and operative rule in physics, then, we propose briefly to examine whether the conformation, organic structure, and physico-chemical phenomena of the human body, seem to indicate an adaptation, or conformity, to the known laws of the electric fluid, in its variously modified forms of action. Allow me, however, in advance, to remark, that, at the present day, to doubt the physical agency of light, heat, electricity and molecular attraction, in controlling the elements and determining the functions of organized living bodies, is to overlook the palpable analogies of nature, and defy the latest and clearest conclusions of experimental philosophy. What candid observer can fail to recognize in the vegetable and animal economy, the presence and activity of the same laws which govern the inorganic kingdoms, only modified and made subordinate for the time being, to the supremacy of the vital principle? Light, acting upon the leaves of vegetables, effects the decomposition of carbonic acid gas and the exhalation of oxygen, after the same manner that in the presence of chlorine, in inorganic bodies, it liberates the same gas by the decomposition of water. Light is refracted too by the lenticular structure of the human eye, just as by a glass lens of similar curvature. Caloric is radiated and absorbed by plants and animals as well as by inorganic bodies. Electricity finds a ready transmission along the living vegetable and animal tissues, as though they were lines of iron or copper wire, and decomposes the circulating salts of the blood, just as it effects analogous changes in saline solutions beyond the dominion of life; while the laws of chemical affinity are continually active in maintaining or destroying particular aggroupments of molecules, often different, it is true, from the inorganic arrangements of the same elements, but dependent solely upon their previous existence and properties for all their new phenomena; for in the appropriate language of Liebig, "all the organs together cannot generate a single element, carbon, nitrogen or a metallic oxide." Surely then we are authorized to commence the survey we have intended.

1st, then: *Electricity may be generated by the friction of solids, or of solids and fluids.*

This well-known law is exemplified among solids, by rubbing together two electrics—as, silk and glass—cat's fur and sealing, wax, etc. May not the warmth produced by brisk friction of the hands together, depend upon the calorific energy of the electricity thus evolved from the non-conducting cuticle—and its immediate absorption and diffusion through the humid subcutaneous tissues? In advocacy of this view, it may be stated that if the epidermic surface be moist, and therefore unfavorable to electric accumulation, heat cannot be generated, or feebly, at best; just as the bits of wood used amongst some of the least civilized nations for kindling their fires, must exhibit dry surfaces before ignition is effected.

But, again, let a portion of mercury be included in a thin glass tube, a foot long, and well secured by a cork—then shaken briskly from end to end—the cork removed, and the tube presented to an electrometer, and it will give signs of strong electrical excitement. Now does not the whole sanguiferous circulation, by a kindred process, contribute to the supply of the normal amount of electricity which the animal body requires, in the constant friction of its fluid mass against the sides of the tortuous cartilaginous tubes through which it passes, hurried on by the powerful *vis a tergo* of the great central muscle—resisting in its pulmonary and systematic career the superincumbent atmospheric pressure of more than thirty-two thousand pounds upon a body of ordinary dimensions, while the compressions and concussions which are the necessary results of muscular contractions and expansions, contribute their quota to the general stock? The important discovery recently made in frictional electricity, comes in opportunely to sustain the views here entertained, viz., that globules of water, and of several other fluids, hurried on with great velocity through tubes by the impulsive force of steam or compressed air, evolves electricity freely. Indeed, the most powerful arrangement hitherto known for the production of quantity is a hydro-electric machine, constructed upon the principal above named, by Mr. Armstrong, and exhibited two or three years since at the Polytechnic Institution, in Regent street, London. Such was its power that even paper and wood shavings were inflamed in the course of the spark.

2d. *Electricity is evolved by changes in the condition of bodies from solids to fluids, and from fluids to gases, or the converse, as proved by Harris, Turner, Lavoiser, and La Place and others, and also by chemical combinations and decompositions, as amply illustrated in the experiments of Pouillet on carbonic acid, Wollaston upon the oxidation of electrical amalgum, etc.*

The elements of bodies are known to exercise certain electrical relationships to each other: those which are most highly positive, as carbon, hydrogen, potassium, sodium, etc., exhibiting the most vigorous affinity for those on the negative extreme, as oxygen, sulphur, nitrogen, chlorine, etc. Now these are the very elements which are found to be in most active play in the animal economy, and in the exercise of their affinities might he expected to evolve electrical currents proportionate to the intensity of their chemical action, and we only regret that our limited time will not allow to trace the various chemical reactions incessantly going on among these elementary bodies in the animal organization. It is due, however, to a clear conception of our views that specific reference should be made to some of the widely prevalent and well known operations which are constantly going on in the inorganic as well as the organic kingdoms around us.

The results of this law, then, are seen exemplified on the most magnificent and grand scale in those meteorological changes which mark the varied year. The evaporation going over the whole liquid surface of our globe, by which water assumes an aeriform condition, and again the condensation of of vapor, thus formed, in the higher regions of the atmosphere, into fluids, and solids, as rain and snow, or hail, all serve to liberate immense volumes of the electric fluid. For a similar reason, the sudden and violent changes of chemical condition produced by the molten mineral masses and hot elastic vapors, disgorged from volcanic craters when coming in contact with the cool and open air, generate abundantly those vivid lightnings which have for ages past blazed around the summits of Ætna and Vesuvius during their great eruptions. Now, recent investigations in organic chemistry have disclosed the fact, that large quantities of this fluid are evolved during the most silent and gentle of the vital changes, as in the germination of seeds, and during the respiration of plants through the expanded surface of their leaves and petals. Pouillet has shown "that the surplus electricity arising from a verdant area of 100 yards square, is sufficient to charge a powerful battery." Water is undergoing pergetual decomposition in the process of vegetable nutrition, and Faraday, after patient experiment upon the subject, has announced the opinion that the quantity expended in separating the elements of one single grain of that fluid, would have produced a strong flash of lighting! Thus, under the high laws of animal life and within the pale of the human constitution, similar changes are incessantly going on, attended by similar exhibitions of the electric fluid. Oxygen, entering the circulation by pulmonary endosmosis, unites with the iron of the red blood-globules, and is borne onward as the hydrated peroxide of the metal in solution, until by a votal process it assists in effecting the transformation of the various tissues—absorbs a portion of the carbon—then becomes carbonate of the protoxide, and after reaching in its career, the surface containing oxygen and hydrogen, undergoes another decomposition by which carbonic acid is eliminated and hydrated peroxide of iron again formed to recommence the accustomed circuit. The changes of the ingesta which occur in the animal stomach, and the auxiliary organs of digestion and assimilation, furnish additional sources of electro-accumulation. (*To be continued.*)

